# Controlled decompression alleviates early brain injury in rabbit intracranial hypertension model by regulating apoptosis/necroptosis

**DOI:** 10.1590/ACB360406

**Published:** 2021-05-28

**Authors:** Can Zhang, Yue Wang, Junhui Chen, Shuo Yang, Yuhai Wang

**Affiliations:** 1 MM. Department of Neurosurgery - Wuxi Medical College of Anhui Medical University - 904th Hospital of PLA (101th Hospital of PLA) - Wuxi, and Department of Neurosurgery - The Second People’s Hospital of HeFei - Anhui, China.; 2 MM. Department of Neurosurgery - Wuxi Medical College of Anhui Medical University - 904th Hospital of PLA (101th Hospital of PLA) – Wuxi, China.; 3 MD. Department of Neurosurgery - Wuxi Medical College of Anhui Medical University - 904th Hospital of PLA (101th Hospital of PLA) – Wuxi, and Department of Neurosurgery - Renmin Hospital of Wuhan University - Wuhan, China.; 4 MM. Department of Neurosurgery - Wuxi Medical College of Anhui Medical University - 904th Hospital of PLA (101th Hospital of PLA) – Wuxi, China.; 5 MD, PhD. Department of Neurosurgery - Wuxi Medical College of Anhui Medical University - 904th Hospital of PLA (101th Hospital of PLA) – Wuxi, China.

**Keywords:** Decompressive Craniectomy, Intracranial Hypertension, RIP, TBI, Necroptosis, Rabbits

## Abstract

**Purpose:**

To evaluate the effects of controlled decompression and rapid decompression, explore the potential mechanism, provide the theoretical basis for the clinical application, and explore the new cell death method in intracranial hypertension.

**Methods:**

Acute intracranial hypertension was triggered in rabbits by epidural balloon compression. New Zealand white rabbits were randomly put into the sham group, the controlled decompression group, and the rapid decompression group. Brain water content, etc., was used to evaluate early brain injury. Western blotting and double immunofluorescence staining were used to detect necroptosis and apoptosis.

**Results:**

Brain edema, neurological dysfunction, and brain injury appeared after traumatic brain injury (TBI). Compared with rapid decompression, brain water content was significantly decreased, neurological scores were improved by controlled decompression treatment. Terminal deoxynucleotidyl transferase dUTP nick end labeling (TUNEL) staining and Nissl staining showed neuron death decreased in the controlled decompression group. Compared with rapid decompression, it was also found that apoptosis-related protein caspase-3/ tumor necrosis factor (TNF)-a was reduced markedly in the brain cortex and serum, and the expression levels of necroptosis-related protein, receptor-interacting protein 1 (RIP1)/receptor-interacting protein 1 (RIP3) reduced significantly in the controlled decompression group.

**Conclusions:**

Controlled decompression can effectively reduce neuronal damage and cerebral edema after craniocerebral injury and, thus, protect the brain tissue by alleviating necroptosis and apoptosis.

## Introduction

Severe traumatic brain injury (sTBI) is a common condition in neurosurgery. According to the epidemiological statistics, the incidence of sTBI is considerably high, which can account for about 10–20% of the total number of surgical traumas, and has a higher wartime incidence^1^,^2^. The increased intracranial pressure (ICP) is one of the main causes of death after sTBI^3^,^4^. The standard decompressive craniotomy is currently the conventional surgery for sTBI in neurosurgery, since it rapidly decreases ICP to minimize brain damage^5^. However, a previous study has associated traditional standard decompressive craniotomy with the occurrence of several complications, such as intraoperative acute encephalocele and postoperative cerebral ischemia^6^. A study reported that conventional standard decompressive craniotomy can lead to hyperemia and over perfusion after cerebral ischemia^7^. Therefore, more research is required on how to improve the surgery to reduce the incidence of complications.

Controlled decompression is a form of craniotomy that slowly releases ICP during surgery (not rapidly released with conventional standard decompressive craniotomy). This allows for gradual brain tissue reperfusion, thereby protecting cerebral vascular and neurological function^8^. This new randomized, controlled trial (ChiCTR-TCC-13004002) showed that controlled decompression surgery can significantly improve clinical outcomes and reduce the postoperative complications of sTBI^9^.

The primary cause of secondary damage in sTBI in neuron death. As a passive process of cell death, necrosis was for a long time considered to be uncontrollable^10^. In 2005, Degterev *et al*.^11^ found that neurons have a controllable type of programmed necrosis, known as necroptosis. Receptor-interacting protein 1 (RIP1) and receptor-interacting protein 3 (RIP3) can activate the signaling cascade under the stimulus of the death signal and cause cell necrosis. This finding is supported by necrostain-1 (NEC-1), a specific inhibitor of RIP1^12^,^13^. Necroptosis is common in ischemia-reperfusion injury and may be an effective mechanism of ischemia-reperfusion injury^14^,^15^.

The objective of this study was to evaluate the effects of controlled decompression and rapid decompression, explore the potential mechanism, and provide the theoretical basis for the clinical application, and explore the new cell death method in intracranial hypertension.

## Methods

### Experimental animals

The Bioethics Committee of the Jiangnan University (JN. No. 20190630R0480810[193]) approved the animal procedures used in this study. Procedures were performed following the National Institutes of Health guidelines for handling and care of laboratory animals.

New Zealand White rabbits (weighing 2.2–2.5 kg, 3 months old) were obtained from the Animal Central of Taihu Hospital (Wuxi, China). They were maintained at a normal level of atmospheric humidity room with a constant temperature (22 °C) and a 12 h photoperiod for at least 15 days before the experiment. They had free access to food and water. Rabbits without anomalies were selected, fasted for 12 h, and used for the experiment.

### Establishing intracranial hypertension model rabbit

**Figure 1:**
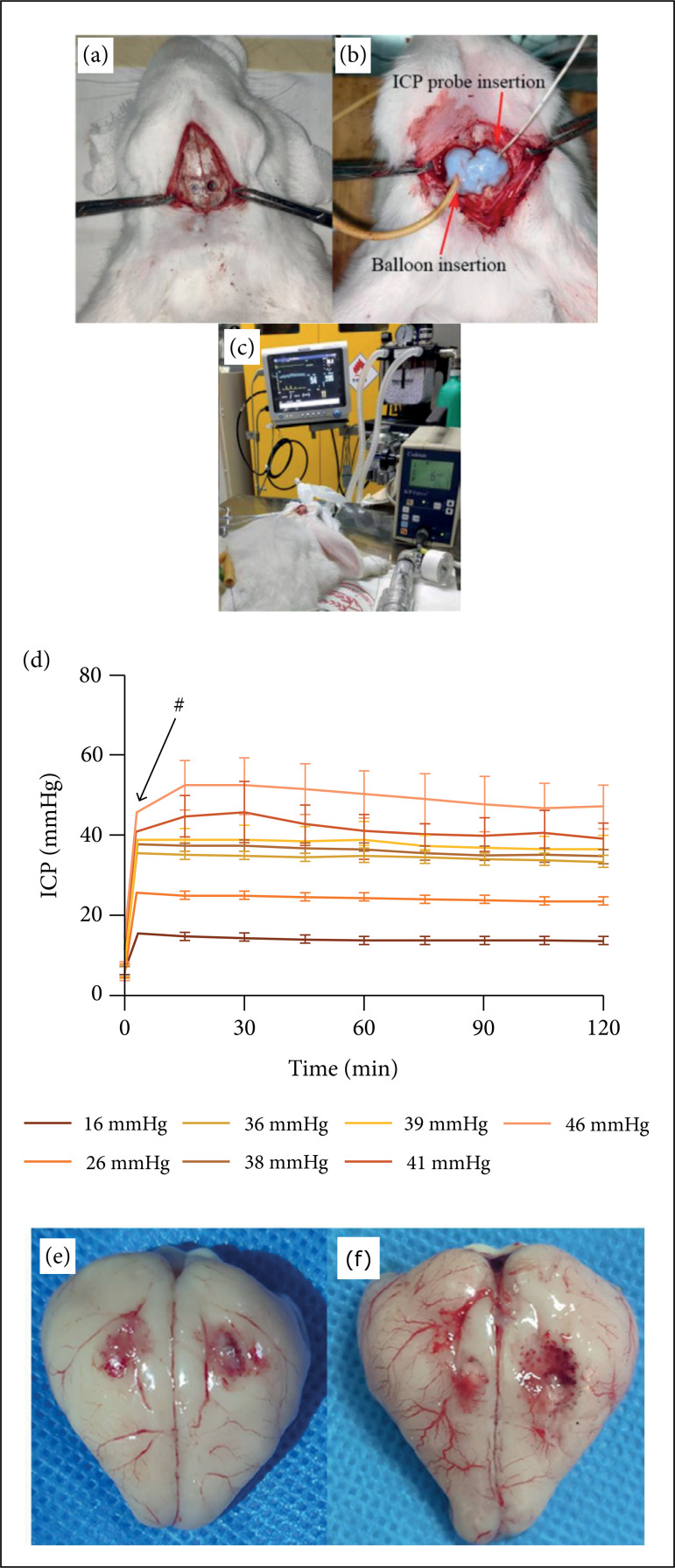
**(a-c)** The process of establishing the model of intracranial hypertension; **(d)** ICP changes in each group within 120 min. (# represents that each group has completed the injection of water into the balloon within 3 min); **(e)** Gross anatomy of the controlled decompression group; **(f)** Gross anatomy of the rapid decompression group Red arrow indicated damaged area.(

The protocol of intracranial hypertension model rabbit was described as a previous study^16^. After 12 h of fasting, the rabbits were anesthetized with intramuscular ketamine (50 mg·kg^–1^), and continuous intravenous infusion of sodium pentobarbital (10 mg·kg^–1^·h^–1^) via the marginal ear vein throughout the remainder of the experiment. The rabbits were placed in a prone position and fixed on the operating bench after being anesthetized. The hair on the head was shaved and the skin on the operation area disinfected. The scalp was then cut open at the center of the head and the skull was dissected. Using a dental drill, bone holes with a diameter of 0.3 cm were drilled behind the coronal suture and 0.5 cm on the left and right sides of the sagittal suture. Bone residues were removed carefully to prevent the exposure and damage of dura mater and blood vessels. The ICP express was inserted into brain tissue about 1.0 cm deep in the left vertical direction. The epidural pressure balloon was placed in the right frontotemporal direction (Fig. 1 a–c). The ICP probe was connected to an ICP monitoring device (Codman, Johnson and Johnson Medical Ltd, 82-6635, USA) to observe ICP changes during operation. An epidural compression balloon was connected to an intracranial infusion pump (Merit Basix Touch Inflation Device, IN4130, USA).

### Experimental design

To explore the limit of ICP, 10 mmHg was increased in each group according to the standard ICP (6 mmHg) of New Zealand rabbits. The ICP was increased to the corresponding value 3 min into the experiment. Intracranial pressure, heart rate, respiratory, and mortality values were observed after 120 min. When most of the experimental animals exhibited a sharp ICP fluctuation (greater than or equal to 15% of the original ICP value) or a significant increase in mortality within 24 h, the ICP threshold was assumed to have been reached. Dynamic monitoring of ICP, the average of the ICP value that reached the ICP limit and the ICP value that did not reach the limit in the previous group were used as the ICP setting value of the next group (take an integer). The ICP value approaching the limit according to the actual situation was divided into 7 groups: (16, 26, 36, 38, 39, 41 and 46 mmHg). There were four rabbits in every group.

The animals were divided into three groups (n = 8/group) after the verification of the ICP limit value. The sham group, which did not inject liquid after placing the epidural pressurized balloon and keeps it for 30 min. In the controlled decompression group, after placing the epidural pressurized balloon, the ICP was increased to 38 mmHg within 10 min and maintained for 30 min. The pressure infusion pump was then used to make the ICP decrease uniformly to a normal ICP level within 30 min. In the rapid decompression group, the treatment was the same as in the controlled decompression group, but the ICP was released much faster than in the controlled decompression group, within 10 s. The balloon and ICP probe were removed, then bone wax and dental resincement (Clearfil SA Cement, RH Dental, Denmark) were used to seal the bone holes and suture the scalp. After recovery from anesthesia, the rabbits were released into the feeding room. All the rabbits were euthanized with 100 mg·kg^–1^ of intravenous pentobarbital injections after 24 h of observation in the feeding room. The cerebral cortex under balloon compression (about 5 mm^2^) was removed immediately after brain dissection on ice for western blot detection and Nissl staining. The other portion was used to estimate brain tissue edema.

### Neurological scoring

An independent observer who was blinded to the research recorded neurological scores 24 h after surgery. A previously modified neurological scoring table (Table 1) was used to assess the neurological function post-operation^17^,^18^.

**Table 1:** Neurological scoring among the three experimental groups.

Category	Behavior	Score
Appetite	Finished meal	0
	Left meal unfinished	1
	Uneaten	2
Activity	Active, squeaking or standing	0
	Lying down, will stand and walk with some stimulation	1
	Almost always lying down	2
Deficits	No deficits	0
	Unable to walk due to ataxia or paresis	1
	Impossible to walk and stand due to ataxia and paresis	2

### Brain water content

The standard wet-dry method was used to compute the brain water content, as previously reported^19^,^20^. The entire brain tissue was taken for water content detection immediately after the animals were killed. After dissection, the brain tissue was promptly placed on a precision electronic scale with tinfoil to record the wet weight (M); the brain tissue was then packaged using tinfoil and put into a drying oven at 110 °C for 24 h. The tissue was weighed to measure the dry weight (m) after restoring the temperature to room temperature. Brain water content = (M − m)/M × 100%.

### Cytokine tumor necrosis factor (TNF)-a measurements

TNF-a was measured by ELISA (Thermo Fisher, A356015) according to the manufacturer’s instructions.

### Nissl staining

Nissl staining was performed as described in the previous study^21^. Briefly, the cerebral cortex was post-fixed in 4% paraformaldehyde at 4 °C overnight, dehydrated and embedded in paraffin, and sectioned (4 μm). The paraffin sections were torrefied at 60 °C for 30 min then dewaxed using xylene. Gradient alcohol dehydration was then followed by slow flushing with running water for 1 min. The sections were then stained with toluidine blue at room temperature for 15 min. After washing slowly with running water, 1% hydrochloric acid alcohol to differentiate, then washed again by running water. The sections were then counterstained in a saturation lithium carbonate solution for 30 s. Finally, they were dehydrated with alcohol, transparentized by xylene, and sealed with gum. An Olympus light microscope (Olympus Corporation, Tokyo, Japan) and Image-Pro Plus 6.0 Software (Media Cybernetics, Inc., Rockville, MD, USA) were used to determine the numbers of Nissl bodies in neurons.

### Measurement of apoptosis by terminal deoxynucleotidyl transferase dUTP nick endlabeling (TUNEL) staining

Apoptosis in neurons was determined using a standard TUNEL staining method according to the manufacturer’s protocol (Roche, Penzberg, Germany).

### Double immunofluorescence staining

The basic methods of double immunofluorescence staining are described in previous studies^19^,^20^. The necroptosis in the rabbit cerebral cortex was evaluated by RIP3 and neuronal staining specific marker (NeuN) double immunofluorescence staining. Briefly, the rabbit cerebral cortex was fixed in 4% paraformaldehyde for 24 h at 4 °C, and a 30% sucrose solution was used to dehydrate the sample. The samples were then sectioned to a thickness of 10 μm. Many of the procedures, including Nissl staining, were similar to histologic staining. The primary antibodies, including anti-NeuN polyclonal antibody (1:200, ab128886; Abcam) and anti RIP3 polyclonal antibody (1:500, bs-3551R; Bioss, China), were diluted overnight in PBS at 4 °C. The goat anti-rabbit IgG secondary antibody (1:500; Beyotime, China) was incubated at room temperature for 1 h after washing and rinsing the sections in PBS. It was incubated and covered with DAPI, rinsed again, and covered with glycerol. Fluorescence microscopy (Leica Microsystems, Germany) was used to observe and count the positive cells.

### Western bolt analysis

Western bolt analysis was used to determine the levels of RIP1, RIP3, and caspase-3 proteins and performed as described previously. The cerebral cortex sample from the left compression area was collected and homogenized to extract protein. Samples were then mixed with sodium dodecyl sulfate-polyacrylamide gel electrophoresis (SDS-PAGE) loading buffer, heated at 100 °C for 5 min, cooled on ice, and centrifuged for 5 min to remove the insoluble precipitate. Samples with a maximum sample size of 20 μL per pore were separated by 10% SDS-PAGE and transferred to a nitrocellulose membrane (Bio-Rad, USA). Rabbit antibodies against RIP1 (bs-5805R; Bioss; China), RIP3 (bs-3551R; Bioss; China), caspase-3 (ab44976; Abcam; USA) were probed with the membrane and overnight at 4 °C. HRP-conjugated goat anti-mouse IgG secondary antibodies (1:1000; Sigma) were added to the membrane after rinsing with TBST and incubated for 2 h at 37 °C. Super-GL ECL hypersensitive luminous solution was used to detect chemiluminescence, and the X-ray film was exposed. The dried film was eventually photographed with the gel imaging analysis system (Gel-Pro Analyzer software [Media Cybernetics, Inc.]) after the development and fixing processing.

### Statistical analysis

All the data were presented as the mean ± standard deviation, and analysis was done in SPSS (version 23; IBM, New York, USA). One-way analysis of variance and least significant difference (LSD) were used for multiple comparison tests. Statistical significance was set at p < 0.05.

## Results

### Mortality and ICP setting

In this experiment, the mortality of the controlled decompression group, rapid decompression group, and the sham group were 11.1% (1/9), 20% (2/10), and 0% (0/8). Additionally, the reasons for animals’ death after surgery can be seen in Table 2. Besides, in the pre-experiment phase, it was observed that when the ICP of the experimental animals rose above 38 mmHg: 58.3% of the ICP of experimental animals fluctuated sharply; apnea, deep breath, and pulmonary signs, such as apparent wet crackles, occurred in 41.7% of the experimental animals; 25% of the experimental animals died within 24 h after surgery, and 16.7% of the experimental animals died during surgery. Thus, the limit of ICP was set to 38 mmHg in the formal experiment (Fig. 1d). To maintain the number of animals in each group, the dead rabbits were replaced during the model establishment process. The compression area of the control and rapid decompression group collapsed more than the compression area of the control group. The red arrow shows the spot hemorrhage. There was no difference on the contralateral side (Fig. 1e).

**Table 2:** Cause and number of deaths.

Group/Cause of death	16	26	36	46	41	39	38
	(mmHg)						
Subdural hematoma	0	0	0	0	0	0	0
Epidural hematoma	0	0	1	2	1	1	1
Respiratory and circulatory disorders	0	0	0	3	1	1	0
Unknown reason	0	0	0	0	0	1	0
Total number of deaths	0	0	1	5	2	3	1

### Controlled decompression improves neurological scoring and brain water content

**Figure 2:**
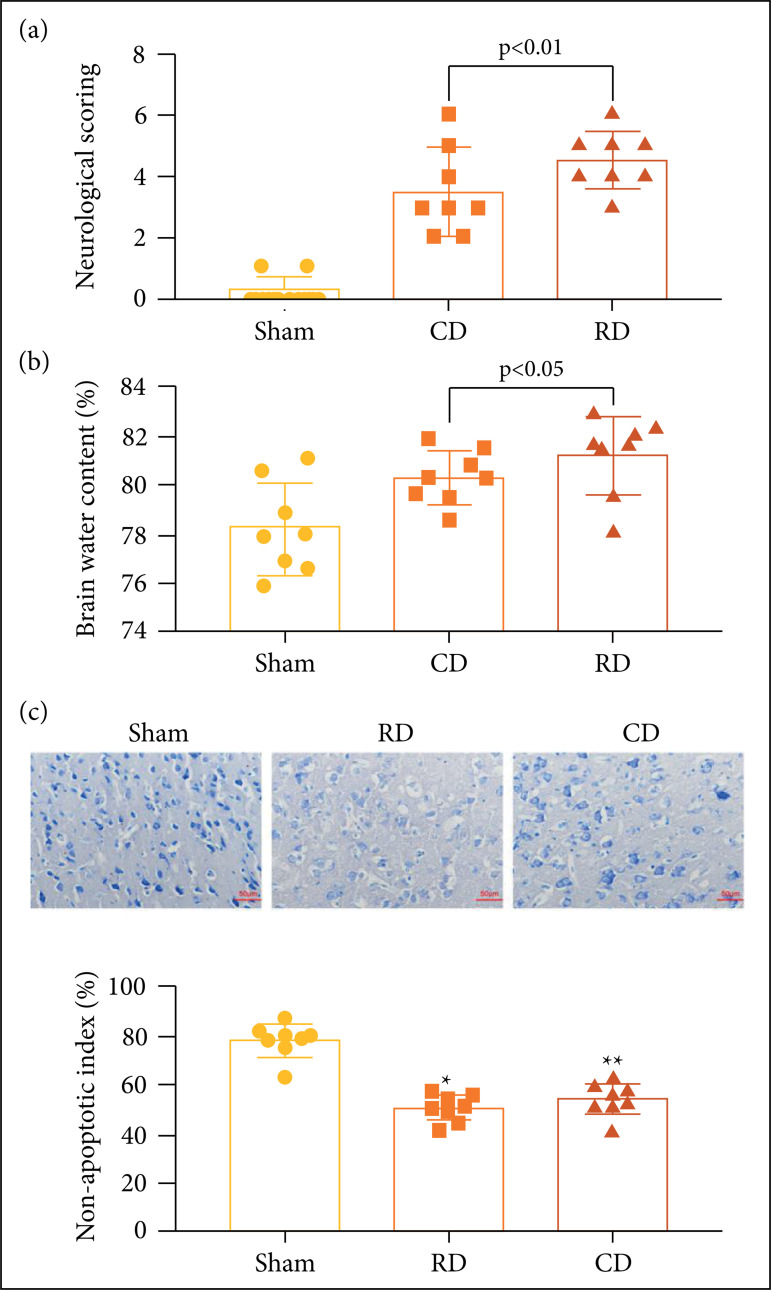
Controlled decompression decreases the neurological scoring and brain water content after surgery. **(a)** The results indicated that neurological scoring was significantly lower in the sham group compared with the rapid group and the control group. The sham group *vs*. the control group and the rapid group, p < 0.01; the control group *vs*. the rapid group, p < 0.01. **(b)** Graphs showing the brain water content of the three groups. The sham group *vs*.the control group and the rapid group, p < 0.05 and p < 0.01; thecontrol group *vs*. the rapid group, p < 0.05 (n = 8, mean ± standard deviation (SD), one-way analysis of variance). **(c)** Nissl staining of cortex following different surgical methods. The neurons were sparsely distributed and disorderly arranged in the compression area of cortex in the rapid group and the control group compared with the sham group, and significant variance in several positive cells of Nissl body was observed in the rapid group and the control group, the rapid group are obvious vacuoles around the cells, and the staining of Nissl body became shallow. For statistical analysis, no significant statistical difference in the number of Nissl body-positive cells (number/100 mm^2^) in the control group compared with the rapid group; *p < 0.01 *vs*. sham group, **p < 0.05 *vs*. sham group (n = 8).

The neurological scoring of animals in the controlled decompression group and the rapid decompression group increased significantly compared with the sham group at 24 h after surgery (p < 0.01; Fig. 2; according to ANOVA). Moreover, neurological scoring in the control group decreased significantly compared with the rapid group at 24 h after surgery (p < 0.01; Fig. 2a; according to ANOVA). The brain water content of the control group and the rapid group were significantly increased (80.5 *vs*. 79.4, p < 0.05; 81.5 *vs*. 79.4, p < 0.01) compared to the Sham group at 24 h post-operation. The brain water content in the controlled decompression group decreased significantly compared with the rapid decompression group (80.5 *vs*. 81.5, p < 0.05) (Fig. 2b).

### Controlled decompression alleviates neuron apoptosis

**Figure 3:**
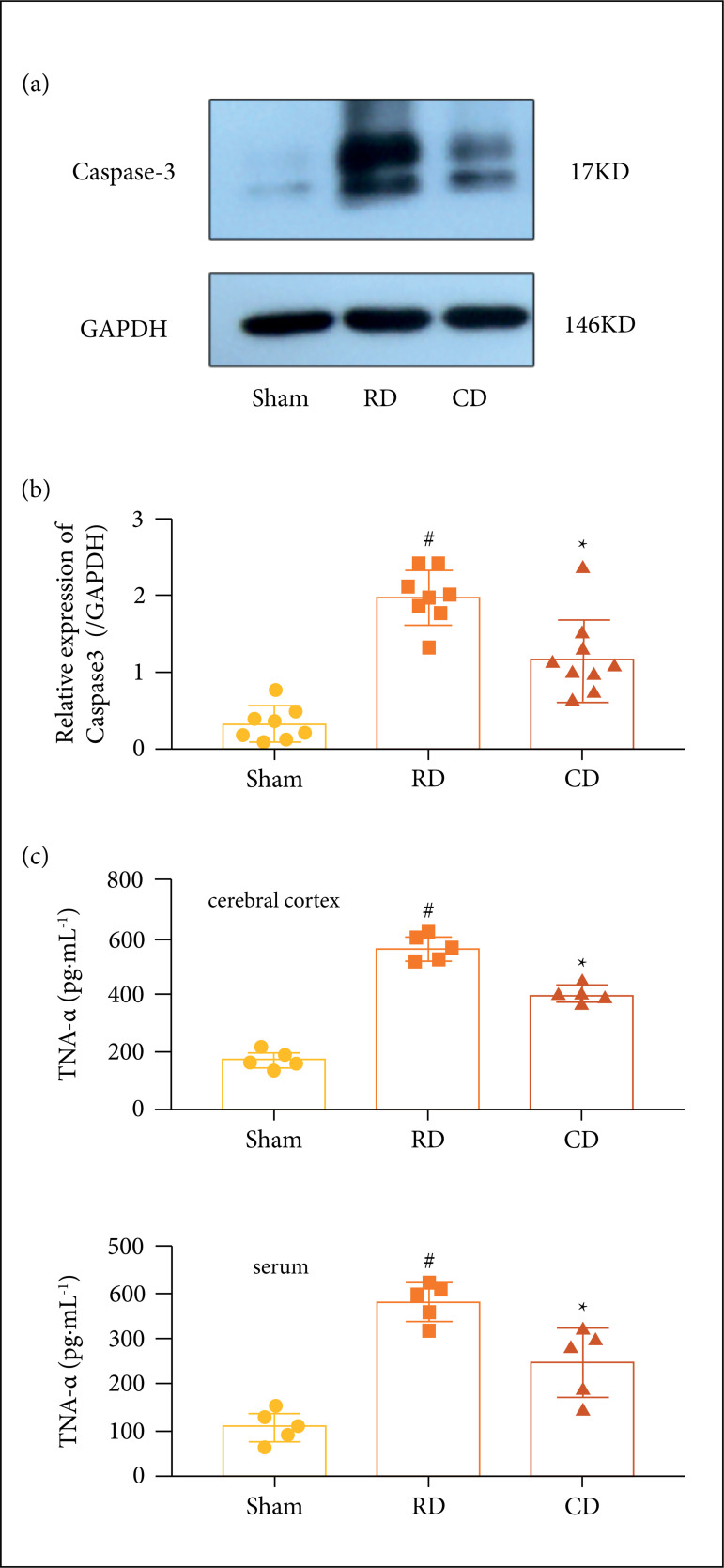
Controlled decompression decreases the apoptosis-related protein expression in the cortex and serum. **(a)** Representative western blot of caspase-3 protein expression. Caspase-3 protein expression in the Sham group was significantly lower than the control group and the rapid group (n = 8/group). Caspase-3 expression levels were significantly decreased in the control group. **(b)** The control group decrease the levels of TNF-a in the cerebral cortex by ELISA (n = 5). **(c)** The control group decrease the levels of TNF-a in the serum by ELISA (n = 5) *p < 0.01 *vs*. sham group, ^#^p < 0.01 *vs*. sham group and rapid decompression (RD) group.

**Figure 4:**
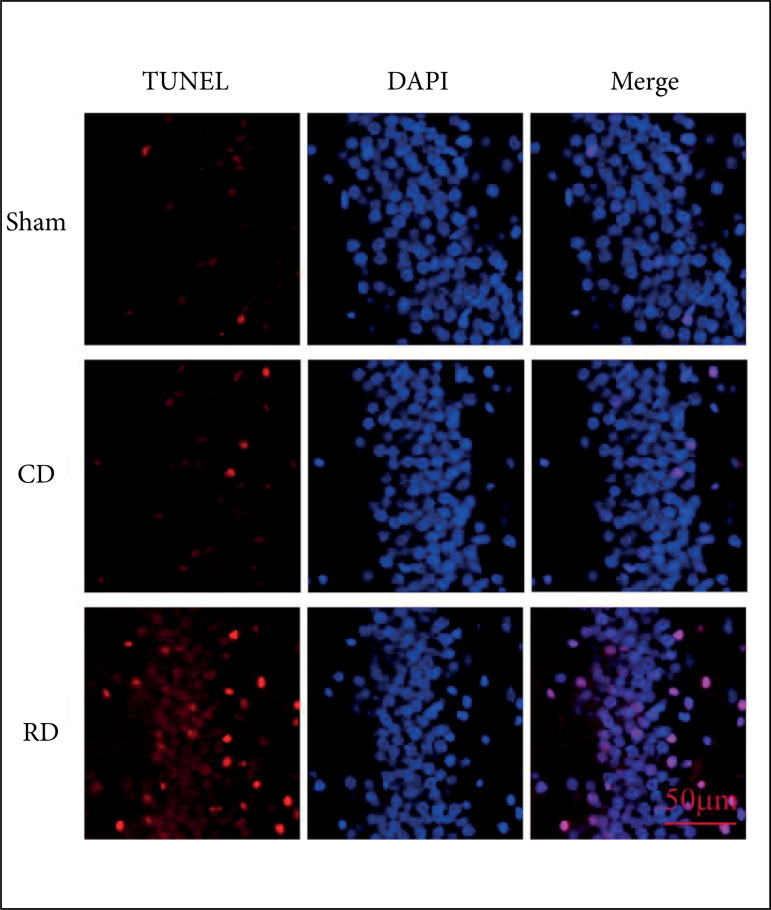
Controlled decompression decreases the neuron apoptosis by TUNEL staining in the hippocampus. It showed that controlled decompression can inhibit neuronal apoptosis compared with the rapid group; apoptosis neuron is enhanced after intracranial hypertension and decreased after controlled decompression.

The image of Nissl staining shows that cortical nerve cells in the rapid decompression group and the controlled decompression group are sparsely distributed and disorganized compared with the sham group. Compared with the controlled decompression group, the rapid decompression group had visible vacuoles around the cell body, and the staining became lighter. The number of positive cells of Nissl body in the controlled decompression group and the rapid decompression group increased significantly compared with the sham group at 24 h after surgery (p < 0.01). However, there was no significant difference in the number of positive cells of Nissl body between the controlled decompression group and the rapid decompression group (p > 0.05) (Fig. 2c). Western blot (WB) was used to detect caspase-3 protein expression levels in the cortex. To observe the apoptosis at 24 h after surgery, the rapid group induced a notable increase of caspase-3 expression levels in the cortex compared with the sham group, whereas the level of caspase-3 was substantially decreased in the control group. Furthermore, compared with the rapid group, the control group decreased significantly (Fig. 3a). It was also found that the control group decreased the TNF-a expression levels significantly in the serum (Fig. 3c) and cerebral cortex (Fig. 3b). The TUNEL staining showed that hippocampus neuron apoptosis in the rapid decompression group and the controlled decompression group were increased compared with the sham after surgery. Besides, in the controlled decompression group, the neurons apoptosis decreased compared with the rapid decompression group (Fig. 4).

### Controlled decompression alleviates neuron necroptosis

**Figure 5:**
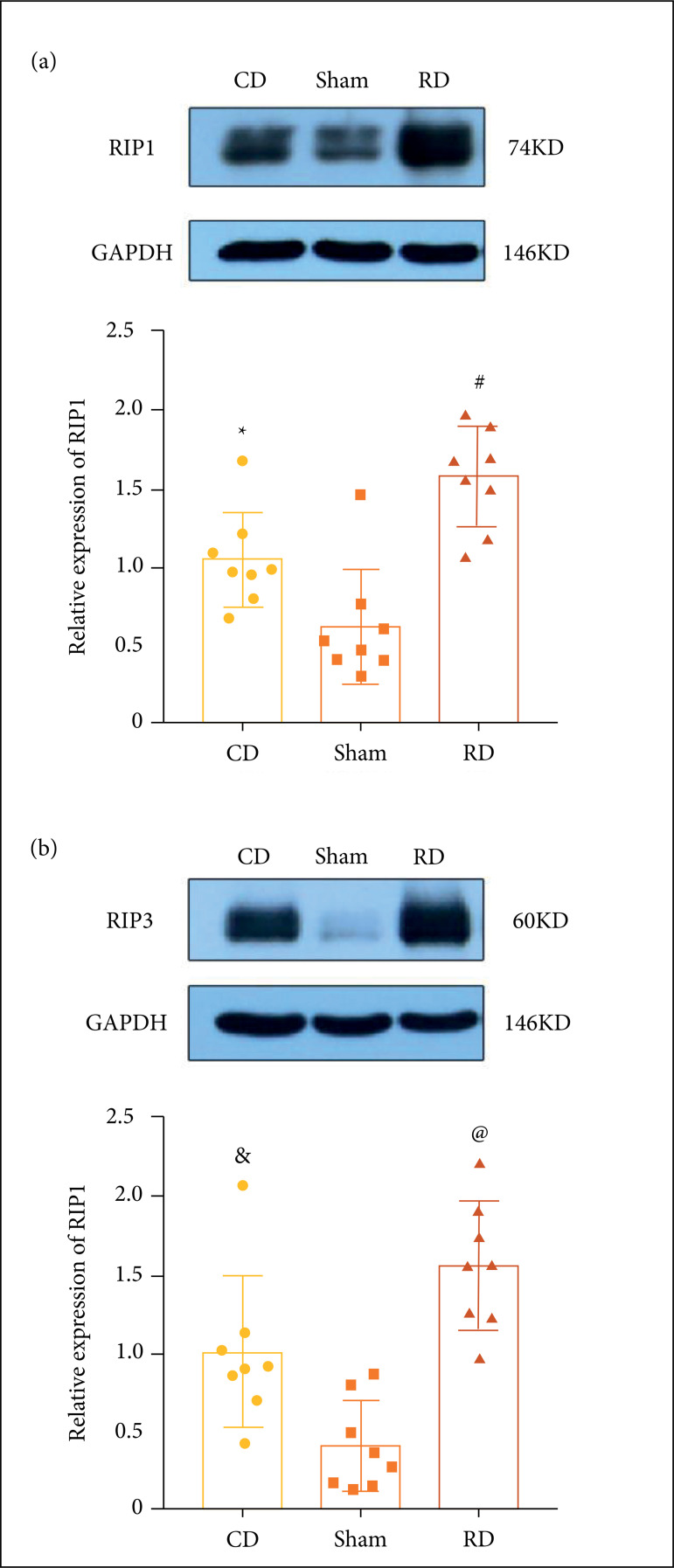
Controlled decompression decreases the expression of RIP1 and RIP3 by western blot (WB). **(a)** Representative WB of RIP1 protein expression. *p < 0.05 *vs*. sham group, #p < 0.01 *vs*. sham group and Controlled decompression (CD) group. **(b)** Representative WB of RIP3 protein expression. &p < 0.01 *vs*. sham group, @p < 0.01 *vs*. sham group and CD group. (n = 8).

**Figure 6:**
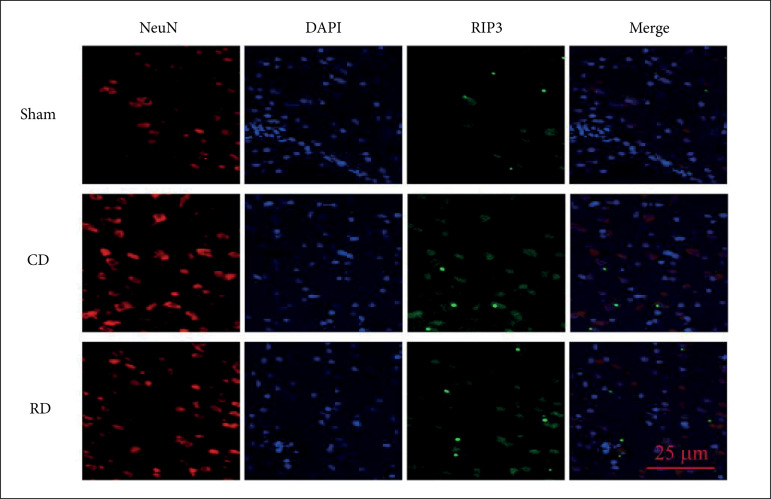
Controlled decompression decreases the expression levels of RIP3. Immunofluorescent staining of RIP3/NeuN/DAPI in the cerebral cortex indicated that controlled decompression can inhibit the expression of RIP3; RIP3 immunoreactivity is enhanced after intracranial hypertension and decreased after controlled decompression.

In this study, WB was used to assess RIP1 and RIP3 expression levels in the cerebral cortex 24 h after surgery. The expression of RIP1 and RIP3 in the controlled decompression group (RIP1: p < 0.05; RIP3: p < 0.01) and the rapid decompression group (RIP1: p < 0.01; RIP3: p < 0.01) significantly increased compared to the expression levels of RIP1 and RIP3 in the sham group. However, the expression levels of RIP1 (p < 0.01) and RIP3 (p < 0.05) in the controlled decompression group were significantly lower than in the rapid decompression group (Fig. 5). After intracranial hypertension induction, double immunofluorescent staining showed that RIP3-positive cells were widespread. Besides, more RIP3 positive neurons were observed in the rapid decompression group compared with the controlled decompression treatment group (Fig. 6).

## Discussion

In this study, the mortality rate, brain water content, and neurological scores were assessed. Nissl staining was also used to evaluate neuron death in three surgical modes. Western blotting and double immunofluorescence staining were eventually used to determine the protein levels of RIP1, RIP3, and caspase-3. These experimental results undoubtedly confirmed that controlled decompression can effectively reduce ICP and inhibit the necroptosis pathway as well. Besides, previous clinical trials also improved surgical patient prognosis and decreased the incidences of postoperative complications^8^,^9^.

Severe traumatic brain injury remains the primary cause of mortality in adults worldwide and is currently one of the biggest public health concerns. Moreover, sTBI complications are still common. The best approach to help these patients could be the application of decompressive craniectomy^22^ with dural augmentation^23^. It has the advantages of full decompression, providing more and larger intracranial buffer space for brain tissue under high pressure, reducing ICP, rebuilding cerebral blood flow perfusion, and controlling secondary brain damage. In the surgical procedure, rapid decompression and rapid removal of hematoma are usually adopted to reduce ICP, which, in turn, increases cerebral blood perfusion and decreases secondary brain injury as well. Although some studies have shown that DC can substantially reduce ICP in patients with severe brain injury, the incidence of complications, including cerebral infarction, remains high. Similarly, morbidity and mortality are still as high as 50–70%^6^,^23^. Thus, reducing the incidence of intraoperative and postoperative complications remains a major challenge. Mcleod stated that some of the complications might be attributed to the rapid removal of bone flaps and hematoma during decompressive craniectomy^7^. The rapid decrease of ICP in contralateral brain tissue, and the absence of the impact of pressure tamponade, results in a rapid hemorrhage of ruptured dural arteries or broken plate barriers, and can even cause hemostatic dural rupture bleeding^7^.

Based on the research of Su *et al*., compressed cerebral blood vessels rapidly expand and become congested after rapid decompression, leading to excessive perfusion of cerebral blood vessels^24^. However, if the perfusion of cerebral blood vessels exceeds a certain threshold, it can damage the blood-brain barrier, increase cerebral blood vessel permeability, and cause excessive plasma components leakage, thus aggravating vasogenic cerebral edema and even causing acute brain swelling^24^. Jiang *et al*. also confirmed that low-pressure perfusion can enhance the recovery of spinal cord function after ischemia and alleviate spinal cord injury caused by reperfusion in rats^25^. Tamaki *et al*. also stated that a rapid reduction in ICP can lead to serious adverse changes in hemodynamics^26^. Therefore, to achieve a stable cerebral perfusion pressure, intracranial hematoma should be gradually removed and ICP gradually reduced^26^. Based on the previous studies and clinical experience, Wang *et al*. first reported a modern treatment mode-controlled decompression, which could control the amount of cerebral perfusion, and transform the one-time perfusion into gradually controlled perfusion^8^. It has since achieved excellent clinical effect^8^. Controlled decompression technology can protect nerve cells and function by reducing ischemia-reperfusion damage and maintaining vascular regulation. This clinical study also demonstrated that the 6-months outcomes, the incidence rates of intraoperative acute brain swelling, and delayed intracranial hematoma were significantly better in sTBI patients who underwent controlled decompression than in those who underwent rapid decompression^9^. Rapid craniotomy (opening the skull and dura quickly, without controlled ICP release) causes large amounts of arterial blood to pour quickly into the brain tissue, but without appropriate venous outflow, then lead to brain edema and brain swelling. The exact mechanism of its action is yet to be established. The pathophysiological process of nerve cell damage after traumatic brain injury (TBI), such as apoptosis and necrosis of nerve cells, should be studied. This will help in exploring the potential molecular mechanism of decompression technology and reduce the incidence of postoperative complications and protect brain tissue^27^.

Necroptosis is a new type of cell death with typical necrotic morphology regulated by signal molecules, including RIP1 and RIP3 in cells^28^. Receptor-interacting protein 3 can phosphorylate RIP1, which can form a stable necrosome complex, activate its downstream necrosis kinase, and eventually result in necroptosis, which can be prevented by a small molecule inhibitor NEC-1^13^,^29^. In recent years, a previous study has proven that necroptosis exists in brain tissue neurons, and is correlated with the neurotoxic effect of 24S-Hydroxycholesterol (24S-OHC) on cortical neurons^30^. Liu reported that intraventricular NEC-1 injection can greatly alleviate brain edema and neuron death in mice after craniocerebral injury^31^. Hypoxia in brain tissue can cause Receptor-interacting protein kinases 3 (RIPK3) upregulation. Receptor-interacting protein 1 and RIP3 upregulation aggravate the death of hippocampal neurons. Also, Previous study have shown that induces the processing of cerebral ischemia-reperfusion. Yang *et al*. had demonstrated that, after cerebral ischemia, the expression levels of RIP3 and mixed-lineage kinase domain-like protein (MLKL) in neurons and astrocytes increased and the area of cerebral infarction caused by ischemia was significantly reduced after RIP3 or MLKL gene knockout^32^. Previous studies have established that necroptosis plays a key role in the pathological process of brain tissue injury, which can, to some degree, intensify brain tissue injury. Therefore, it was speculated that controlled decompression technology can, to a certain extent, block necroptosis, reduce neuron death, improve ischemia-reperfusion injury, reduce the infarct area of brain tissue, and reduce brain edema.

Tumor necrosis factor (TNF)-a is implicated in both pathways of apoptosis and necroptosis and also a key cytokine eliciting the early inflammatory cascades in ischemia-reperfusion injuries. In the present study, it was also found that the expression levels of TNF-a can decrease significantly in the controlled decompression group. Controlled decompression technology maybe decreases the release of TNF-a by regulating the pathway of necroptosis and apoptosis. On the other hand, TNF-a can activate the pathway of necroptosis and apoptosis. Li *et al*. also reported that necroptosis plays a vital role in regulating its pro-death kinase activity in response to TNF-α and pro-survival activity in response to toll-like receptors (TLRs) signaling^33^. Yun *et al*. reported that signal transducer and activator of transcription 3 (STAT3) activation in microglia can increase TNF-α expression, then it can increase neuronal apoptosis by increasing reactive oxygen species (ROS) levels in the neuronal cells^34^.

In this study, by comparing the expression of necroptosis markers under three treatment modes, the expression of RIP1 and RIP3 was found to be significantly reduced under the controlled decompression mode. Therefore, it was concluded that controlled decompression technology can alleviate ischemia-reperfusion injury by regulating the necroptosis pathway. The apoptosis pathway was also evaluated by assessing the expression level of caspase-3, which was activated and overexpressed in the TBI model. The caspase-3 inhibitor can reduce the apoptosis of neurons^35^. Mazumder *et al*. indicated that caspase-3 is an important factor in the apoptosis process^36^. This study showed that caspase-3 in the controlled decompression group was significantly decreased compared with the rapid decompression group. Therefore, a controlled decompression technique can confer neuroprotection after intracranial hypertension injury via regulated necroptosis and apoptosis.

## Conclusion

Compared with rapid decompression, controlled decompression technology can effectively reduce neuronal death after craniocerebral injury, alleviate ischemia-reperfusion injury, and consequently protect brain tissue. Controlled decompression is a new surgical method based on conventional rapid decompression that should be translated into use. The mechanism for minimizing intraoperative and postoperative decompression complications might be by inhibiting the pathway of necroptosis to a certain extent after craniocerebral injury. However, the precise mechanism should be established.
